# Thermal Transitions of Cocoa Butter: A Novel Characterization Method by Temperature Modulation

**DOI:** 10.3390/foods8100449

**Published:** 2019-10-02

**Authors:** Julia Rocha Gouveia, Kelly Cristina de Lira Lixandrão, Lara Basílio Tavares, Paulo Henrique Lixandrão Fernando, Guilherme Elias Saltarelli Garcia, Demetrio Jackson dos Santos

**Affiliations:** 1 Nanoscience and Advanced Materials Graduate Program (PPG-Nano), Federal University of ABC (UFABC), Santo André 09210-580, Brazil; kellycrislira@yahoo.com.br (K.C.d.L.L.); lara.btavares@hotmail.com (L.B.T.); paulo.lixandrao@ufabc.edu.br (P.H.L.F.); demetrio.santos@ufabc.edu.br (D.J.d.S.); 2 Center of Engineering, Modeling, and Applied Science (CECS), Federal University of ABC (UFABC), Santo André 09210-580, Brazil; guilherme.elias@outlook.com

**Keywords:** cocoa butter, thermal transitions, refractive index, TMOR

## Abstract

For the first time, the novel experimental technique Temperature Modulated Optical Refractometry (TMOR) was employed for cocoa butter thermal transitions characterization. The average refractive index (N_MEAN_), the volume (v) change, and the volumetric expansion coefficient ($\beta_{q}$) as well as the dynamic quantities $\beta^{\prime }$ and $\beta^{\prime \prime }$ (real and imaginary volumetric expansion coefficient, respectively) were monitored during cooling and heating and compared to the heat flow curves obtained via the standard technique dynamic scanning calorimetry (DSC). The investigation of these quantities showed that TMOR analysis can yield not only thermal transitions temperatures that are comparable to DSC results, but also some new thermal events that are not detected by DSC. This outcome suggests that TMOR might provide some additional insights on cocoa butter melting and crystallization by means of frequency-dependent measurements due to temperature modulation. This new information that can be accessed during temperature ramps might provide a deeper insight into thermal behavior of fat-based foods, evidencing TMOR value as a tool for thermal transitions investigation.

## 1. Introduction

Chocolate is a dense suspension of solid particles arranged in a complex microstructure. Commercial chocolates are usually composed by approximately 70% of fine particles such as sugar, cocoa powder, and milk solids, which are dispersed in a fat continuous phase, mostly of cocoa butter (CB) [1,2]. Although itself tasteless, CB is a key ingredient in chocolate production, since it is the main structuring material in its composition [3]. Industrially, chocolate is processed in the molten state under specific shear and cooling conditions, aiming to CB solidification in an ordered crystalline structure. In contrast to many other fats, CB is a relatively simple triacylglycerol (TAG), mainly composed of 1,3-dipalmitoyl-2-oleoyl-glycerol (POP), 1,3-distearoyl-2-oleoyl-glycerol (StOSt) and rac-palmitoyl-stearoyl-2-oleoyl-glycerol (POSt) [4]. As a consequence of this composition, CB undergoes solidification in several crystalline polymorphic forms. CB displays a complex structural behavior, despite the simple composition, resulting from the complex polymorphism of the triacylglycerides. This polymorphism is described in the literature and defined by six polymorphic forms, which are classified form I to VI, according to the nomenclature of Wille and Lutton, [5,6,7]. The understanding of the formation of the different polymorphic forms, i.e., understanding the melting and crystallization behavior of CB, is of uttermost importance for chocolate manufacturing, because its composition will directly affect the chocolate final properties, e.g., contraction, snap, gloss, melting properties, and bloom resistance. Therefore, understating melting behavior and crystallization of CB is of uttermost importance for chocolate manufacturing.

The thermal transitions and final morphology of CB has been the topic of many works in recent years [4,8]. Usually, this investigation is performed by thermal-analytical techniques, mainly differential scanning calorimetry (DSC), (nuclear magnetic resonance) NMR and polarized light microscopy (PLM) [9,10,11,12,13]. Although well-established and highly available, DSC analysis does not come without drawbacks, once it relies on the heat transfer between the furnace and the sample through the pan, which might lead to a time lag that brings uncertainties to the time and/or temperature of the occurrence of thermal events.

The novel-experimental technique Temperature Modulated Optical Refractometry (TMOR) emerges as a promising approach to monitor condensed matter behavior and phase transitions under a controlled temperature program [14]. Developed by Anton Paar OptoTec in cooperation with the group of Dr. Krüger, TMOR identifies changes on sample’s refractive index (n) for a wavelength of 589 nm with a relative precision of 10^−6^ [15]. According to Lorentz-Lorenz equation, n can be related to sample density (ρ):

(1) $$\frac{n^{2}-1}{n^{2}+2}=r\;\rho$$

where r is the specific refractivity, accounts for the electronic polarizability of the sample. This quantity can be assumed as constant, once the optical dipole moments depend only weakly on temperature. However, for temperature dependent measurements, small temperature changes (i.e., low temperature rate) must be assured, in order to better fulfill the condition r = constant [16]. As stated in Equation (1), the changes of n due to temperature and/or time mirrors the changes of mass density and, consequently, the volume. The assumption of the Lorentz-Lorenz equation allows one to assess the volume evolution during temperature/time scan ($v\left(t\right)=\rho_{0}/\rho \left(T\right)$, where $\rho_{0}$ is an arbitrary mass density). The volumetric expansion coefficient ($\beta_{q}\left(T\right)$) might be defined as the fractional increase in volume with increase in temperature. Accordingly, TMOR can also yield $\beta_{q}\left(T\right)$, which can be deduced from Equation (2):

(2) $$\beta_{q}\left(T\right)=\;-v^{-1}\partial_{T}v=-\frac{6n\partial_{T}n}{\left[\left(n^{2}+2\right)\left(n^{2}-1\right)\right]}$$

Another key aspect of TMOR is its capacity to impose a sinusoidal temperature modulation to the sample during (i) isothermal, (ii) temperature-ramp, and (iii) temperature steps. The small temperature perturbation, described in Equation (3), induces a refractive index response, which follows the same sinusoidal profile, but with a phase shift between both signals, as presented in Equation (4).

(3) $$T\left(t\right)=\;\bar{T}+\;A^{T}\sin\left(2\pi ft\right)$$

(4) $$n\left(t\right)=\;N_{MEAN}+\;A^{n}\sin\left(2\pi ft-\;\phi \right)$$

where t refers to time, $\bar{T}$ is the reference temperature, $A^{T}$ is the temperature amplitude (perturbation), and f is the frequency of modulation. With respect to Equation (4), $N_{MEAN}\;$ represents the average response, and its calculated using a moving average over one modulation period [16] and obtained from TMOR software. $A^{n}$ is the amplitude of the refractive index, and ϕ is the phase shift between the temperature modulation and the refractive index response. Changes of the phase lag might be associated to relaxation process at the chosen frequency. Based on the dynamic response due to temperature modulation, the complex thermal expansion coefficient can be obtained by merging the Equations (3) and (4) to Equation (2) providing complementary information, that is the dynamic volume expansion coefficient $\beta^{*}\left(f,\bar{T}\right)=\;\beta^{\prime }\left(f,\bar{T}\right)+\;i\beta^{\prime \prime }\left(f,\bar{T}\right)\;$, where $\beta^{\prime }$ accounts for the real part and $\beta^{\prime \prime }$ the imaginary part (Equations (5) and (6), respectively). The detailed description of the technique physical background is rather complex and out of the scope of this work. For more information regarding TMOR, the reader is referred to ref. [15,16]:

(5) $$\beta^{\prime \left(f,\bar{T}\right)}=\frac{6\bar{n}\left(\bar{T}\right)A^{n}\left(f,\bar{T}\right)\cos\left(\phi \left(f,\bar{T}\right)\right)}{A^{T}\left({\bar{n}}^{2}\left(\bar{T}\right)+2\right)\left({\bar{n}}^{2}\left(\bar{T}\right)-1\right)}$$

(6) $$\beta^{\prime \prime \left(f,\bar{T}\right)}=\frac{6\bar{n}\left(\bar{T}\right)A^{n}\left(f,\bar{T}\right)\sin\left(\phi \left(f,\bar{T}\right)\right)}{A^{T}\left({\bar{n}}^{2}\left(\bar{T}\right)+2\right)\left({\bar{n}}^{2}\left(\bar{T}\right)-1\right)}$$

Albeit initially proposed to investigate transparent and isotropic materials, Häupler and Flöter recently employed TMOR technique to investigate the crystallization and melting behavior of palm oil in comparison to conventional DSC analysis [17]. Results revealed systematic differences between the two techniques. TMOR yielded higher crystallization temperatures and lower temperatures for melting. The authors believe that TMOR might provide more accurate results when compared to DSC due to reduction of the thermal lag. This assumption was based in mainly two reasons: (1) Direct contact between sample and prism (in contrast to DSC that requires a metallic pan), and (2) the reduced sample volume, which is approximately 6 mm^3^ for DSC against a contact area of around 1 mm^2^ for TMOR. Considering the before-mentioned aspects, the response of the sample to the temperature change is more direct. Additionally, the possibility to impose a sinusoidal modulation in temperature also represents a significative gain with respect to conventional DSC analysis because it allows to study dynamic effects as well. 

In their sequential effort (21), N-Hexadecane, palmitic acid, and glycerol were the subject of a similar study. Results showed a good agreement of DSC and TMOR data in both melting and crystallization events for all the three substances, following the same systematic difference found in the previous study (i.e., TMOR yielded higher crystallization temperatures and lower melting temperature). The authors acknowledged that although further studies on different system are necessary, TMOR can also be suited for crystalline materials and might be a powerful tool for investigating their behavior under a controlled temperature program. 

In our work, we investigated for the first time the TMOR capacity to identify the melting transitions of CB. To this end, the melting and crystallization behavior of this industrially relevant lipid was investigated by TMOR and compared against the conventional DSC analysis. For that matter, CB was studied by TMOR and DSC under cooling and heating conditions employing the same temperature rate. Results showed that in addition to the thermal transition temperatures that agreed well with DSC, TMOR is also able to probe some other thermal events that are not visible on DSC curves. This outcome highlighted TMOR as an accurate and practical tool to investigate thermal transitions of some edible fats and open a large range of applications for this novel technique.

## 2. Materials and Methods

Cocoa butter was acquired from Cargill Agrícola SA (Brazil). It is a deodorized form with the following main triacylglycerol composition: 19.6% of palmitic-oleic-palmitic (POP), 22.7% of palmitic-oleic-stearic (POSt), and 22.7% of stearic-oleic-stearic (StOSt). TMOR measurements were performed on a TORC 5000 from Anton Paar (UFABC, Santo Andre, Brazil) to evaluate the thermal transitions of cocoa butter in cooling and heating ramps. Around 0.5 g of CB was placed directly on the temperature-controlled prism and the cavity was sealed. Sample was heated to 55 °C and kept at this temperature for 20 min to erase the thermal history. Afterwards, the sample was cooled down to 4 °C with a cooling rate of 0.5 K min^−1^, modulation period of 60 s (f_MOD_ = 17 mHz) and amplitude of 0.5 K. After 8 minutes at 4 °C, the sample was heated again to 55 K with a heating rate of 0.5 °C min^−1^ and same modulation frequency and amplitude (17 mHz and 0.5 K, respectively), and kept at this temperature for 8 minutes. The cooling and heating rate (0.5 K min^−1^, respectively) and the modulation frequency (17 mHz) were chosen based on the parameters reported at [17,18]. 

The DSC curves were recorded with a Q200 equipment from TA Instruments (UFABC, Santo Andre, Brazil). Around 10 mg of CB were placed in aluminum pan and sealed with a pinhole-lid. For the sake of comparison with TMOR results, the same thermal program was employed for this analysis (both cooling and heating in air), with exception of the temperature modulation, which was not available on this standard equipment.

## 3. Results and Discussion

**Figure 1:**
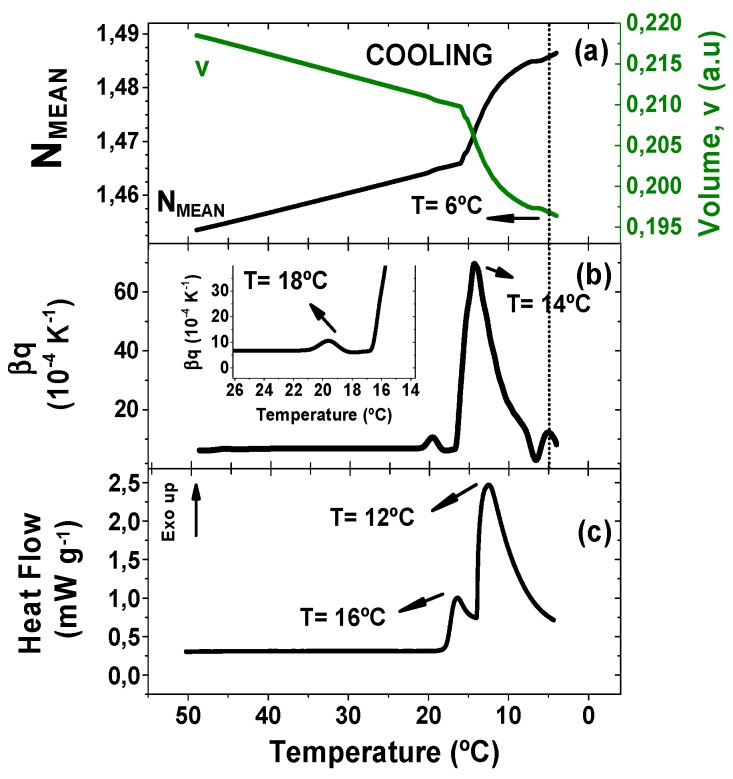
Comparison of the temperature dependence during cooling at 0.5 K min^−1^ of (**a**) NMEAN and Volume (a.u.) recorded via TMOR with temperature modulation of 0.5 K amplitude and 17 mHz modulation frequency. (**b**) The thermal volume expansion coefficient β_q_ (K^−1^) recorded via TMOR with temperature modulation of 0.5 K amplitude and 17 mHz modulation frequency, and (**c**) heat Flow (mW g^−1^) recorded via DSC.

Our efforts in this work were dedicated to investigating the feasibility of TMOR as a tool for thermal analysis of cocoa butter by comparing its results to benchmarked DSC technique. For a fair correlation between the two of them, the same temperature program was imposed in both methods (0.5 K min^−1^). Figure 1 depicts the comparison of the temperature evolution of the quantities probed by TMOR and DSC.

Figure 1 shows the results obtained during cooling process, in which (Figure 1a) the refractive index has a linear increase with the decrease of temperature up to 15 °C. This change accompanied by the decrease of the volume v in arbitrary units is typical for liquids and can be related to densification (see Equation (1)). This linear behavior is followed by a drastic refractive index increase (volume decrease), which might be justified by the formation of crystals. In addition, the temperature dependence of the thermal volume expansion coefficient β_q_, obtained by equation 2, is displayed in Figure 1b. β_q_ initial and final values are slightly different going from 6.23 × 10^−4^ K^−1^ to 8.40 × 10^−4^ K^−1^. This trend is expected, since the thermal expansion coefficient in crystals should be higher than in the melted state [19]. Furthermore, the final value of β_T_ (8.40 × 10^−4^ K^−1^) is in good agreement with the value reported by literature of 7.72 × 10^−4^ mL g^−1^ K^−1^) [20].

The thermal volumetric expansion coefficient behavior during cooling also revealed different events, which might be related to the crystallization and/or polymorphic transformation processes. The first and small peak appears at 18 °C (Figure 1 insert) followed by a strong high and broad peak at 14 °C. Lastly, a new event was found at 6 °C, which mirrors an anomaly found on N_MEAN_ and v (evidenced by the dashed line). DSC curve was recorded at the same temperature program and is shown in Figure 1c. Two crystallization peaks are found at 12 °C and 16 °C. Cocoa butter crystallites are described in the literature in six different polymorphic forms, frequently named as γ(I), α(II), β‘(III), β’(IV), β’(V), and β’(VI), here presented in crescent thermal stability [21]. The different polymorphic forms are formed depending on the heating rate and annealing time. It is out of the scope of this work the exact determination of the polymorphic forms, once a fast and linear cooling ramp was applied to CB in our study. Nevertheless, a comparison between our results to literature results is feasible, considering the physical meaning of the TMOR curves. The peak found at 18 °C and at 16 °C on TMOR and DSC, respectively, might be assigned to the form β‘(IV) that has shown to have a rapid-stating recrystallization from melt at temperature ramp program [22]. The second peak can be associate to the formation of α(II) polymorphic phase at 12 °C and 14 °C accordingly to TMOR and DSC [4]. The third thermal event that was only detected via TMOR might be associated to the formation of γ(I), which has been found to crystallize at low temperatures [22].

Despite the polymorphic phase that was formed, the heat flow peaks (16 °C and 12 °C) are in good agreement with TMOR results (18 °C and 14 °C) with a systematic variation of +2 °C. The same variation trend was observed in Häupler studies of fats crystallization employing TMOR and DSC [18]. Convergence between TMOR and DSC peaks, associated to well-stablished relationship between the peaks at DSC results and crystal formation, allows to relate TMOR peaks with crystallization. Finally, we must emphasize, that DSC analysis did not yield any counterpart for the 6 °C peak found via TMOR. Several works in literature have associated DSC to other techniques, such as polarized light microscopy (PLM) and X-ray diffraction DRX [23,24,25] for studying cocoa butter thermal and phase transitions. Their results confirmed that not every polymorphic transition can be probed by DSC due to the low energy associated to a specific transition or superposition with other thermal events. The use of a modulated temperature DSC (MTDSC) could provide further information on this topic. Analysis carried out in MTDSC also benefit from temperature modulation, same principle involved in TMOR methodology. However, TMOR has a higher precision (ca. 10^−6^) and a more efficient heat transfer associated to the temperature modulation, which offers some advantages on the characterization of different thermal events. Although the determination of the polymorphic form associated to each crystallization event is out of the scope of this work, this result suggests that TMOR might yield new insights on thermal analysis of CB.

At this point an important remark must be done with respect to the comparison between DSC and TMOR results. The first is a well-stablished methodology for thermal properties characterization based on the heat exchange between the sample and a reference pan. Although available, in this work any kind of temperature modulation was imposed during DSC analysis, therefore, we can classify this methodology as a kinetic analysis. On the other hand, TMOR analysis probed CB samples with a temperature modulation of 0.5 K of amplitude and 17 mHz of modulation frequency, yielding dynamic results. Due to the difference in probing modes and quantities, one must bear in mind that correlation between TMOR and DSC must be done in a technological perspective, rather than a physical approach. 

**Figure 2:**
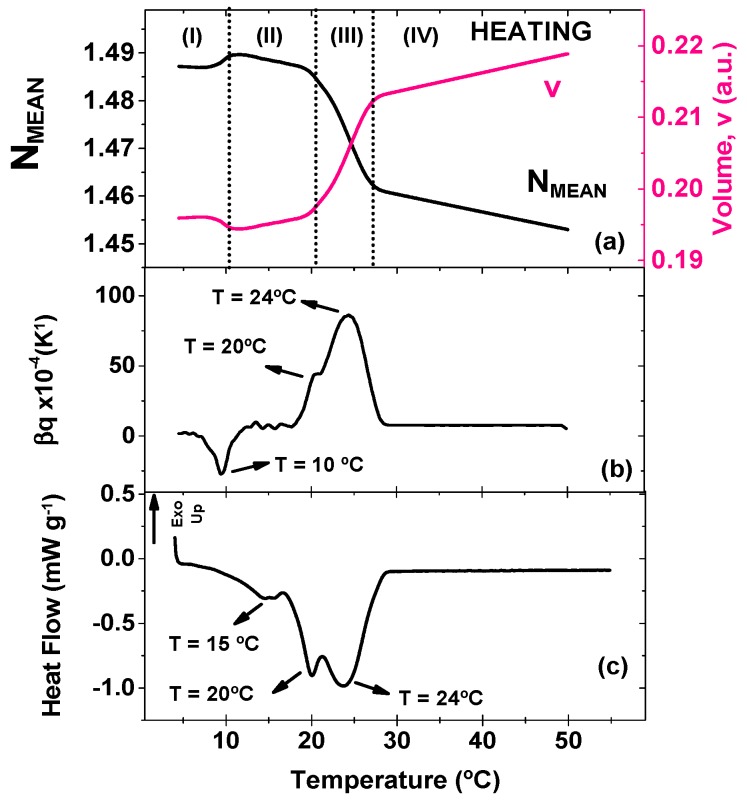
Comparison of the temperature dependence during heating at 0.5 K min^−1^ of (**a**) N_MEAN_ and Volume (a.u.) recorded via TMOR with temperature modulation of 0.5 K amplitude and 17 mHz modulation frequency. (**b**) The thermal volume expansion coefficient β_q_ (K^−1^) recorded via TMOR with temperature modulation of 0.5 K amplitude and 17 mHz modulation frequency, and (**c**) heat Flow (mW g^−1^) recorded via DSC.

A similar plot concerning the heating procedure is displayed in Figure 2 where TMOR and DSC results are compared. N_MEAN_ and v curves show a 4-stage behavior with temperature ramp, as highlighted in Figure 2b. Initially, N_MEAN_ increased slightly accompanied by a decrease in v (stage I, ~10 °C), until it appears as a peak at ~20 °C (stage II), and then decreases abruptly with further temperature increase (stage III, up to 27 °C). At stage IV, CB behaves as a liquid material, confirming the occurrence of complete melting. The slight increase in N_MEAN_ at low temperatures might be associated to the transformation of a metastable crystal to a different phase and/or phase separation. This anomaly is accompanied by a peak on β_T_ curve (Figure 2b), but again no correspondent event is seen on DSC output (Figure 2c). β_T_ curve also gives some insights in terms of the temperature in which the transitions occur. One strong peak appears at 24 °C and a shoulder at 20 °C. These values agree well with DSC curve that shows two endothermic peaks at 20 °C, and 24 ºC. Furthermore, a third peak is found on DSC curve at 15 °C, but no correspondent anomaly is found on β_T_. 

According to literature, the transition at 15 °C is associate to the polymorphic form α(II), while peaks at 20 °C and 24 °C might be assigned to polymorphic forms β’(III) and β’(IV), respectively [3]. Comparing this outcome to the cooling ramp, it is possible to identify an analogue for crystallization and melting temperature for α(II) and β’(IV). On the other hand, the cooling curve suggested the formation of γ(I), that was not seen on the melting curve. At the same time, during heating, a peak that might be assigned to β’(III) was found at 20 °C. This discrepancy amid the two curve strengths our speculation that a polymorphic transition was identified by TMOR at around 10 °C as this temperature is too high for the existence of the unstable γ(I) polymorphic form. Although more investigation must be performed in order to understand the definitive origin of this thermal event at 10 °C, once again TMOR proves its value as a tool for investigating specific microstructural modifications in complex matter.

**Figure 3:**
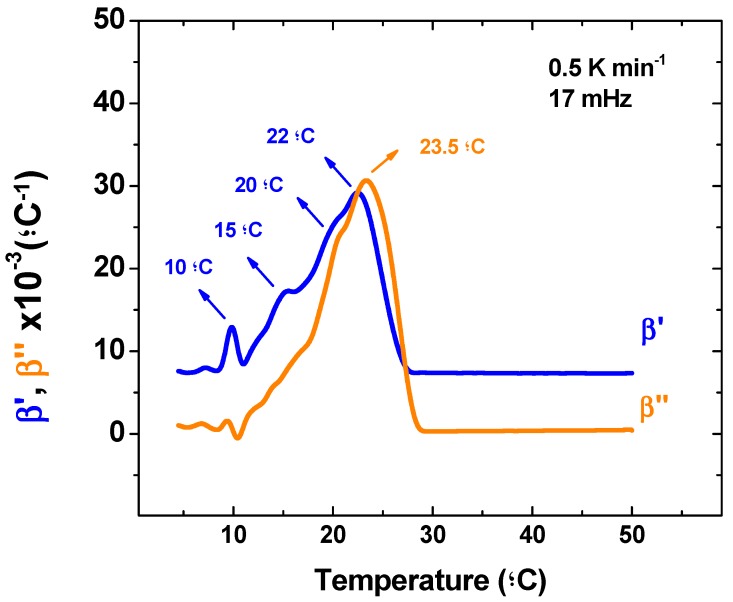
Temperature dependence of the dynamic thermal volume expansion coefficient. β’ (blue line) and β’’ (orange line). These quantities are acquired simultaneously with quantities pictured in Figure 2a,b.

The following discussion considers the dynamic part of the volume expansion coefficient and once more an important remark about TMOR analysis must be made. So far, the only quantities used from TMOR were obtained from the average refractive index N_MEAN_. As evidenced by equations 1 and 2 this measurement does not take into account the temperature modulation and considers only the temperature ramp (see Equations (1) and (2)). For that reason, these quantities (N_MEAN_, v, and β_T_) have been regarded to account for the kinetic part of the transition in temperature ramp [26]. However as stated in the introduction, TMOR is also able to impose a sinusoidal temperature modulation that can yield the dynamic response of the sample. Therefore, TMOR can simultaneously probe the kinetic and dynamic response of the sample. At this point the question arises which other thermal events could be identified by the dynamic behavior of the volume expansion coefficient and for that matter Figure 3 displays β’ and β’’ behavior during melting.

The β’ curve also shows a strong peak at 10 ºC that has probably the same origin as the peak seen in βT. However, at the higher temperature region β’ present a broad peak that is centered at 22 ºC and has clear shoulders at 15 °C and 20 °C. Although the deconvolution of this peak and association to correspondent thermal events is out of the scope of this publication, it is here once again a strong evidence of TMOR ability to investigate thermal transitions, which arise from the possibility of combining both kinetic and dynamic approaches to certain phenomena.

## 4. Conclusions

The thermal behavior of cocoa butter was investigated by TMOR and DSC under cooling and heating. Under cooling regime, the kinetic quantities probed by TMOR, namely N_MEAN_, v, and βT yielded two thermal transitions that were assigned to the crystallization of different polymorphic crystalline forms. The transition temperatures agreed well with DSC results with a systematic variation of 2 °C. Additionally, a third thermal event was detected by TMOR, while no counterpart was found on DSC result. During heating, the dynamic quantities β’ and β’’ acquired due to temperature modulation were investigated along with the kinetic curves. The combination of both approaches (kinetic and dynamic) showed again comparable results to DSC related to the melting temperature of different crystals; with the addition of a low temperature transition that was not perceptible in the heat flow curve and might be related to a polymorphic transition. 

Overall, this study shows that transition temperatures depicted by TMOR during thermal analysis of cocoa butter are comparable to the benchmarked method DSC. Furthermore, the possibility of simultaneously measuring the kinetic and dynamic response has proven to be a powerful tool to resolve hidden thermal transitions that are not detectable with DSC. Therefore, the scientific and technical power of TMOR for gaining insights on thermal transitions of edible-oils is here demonstrated.
